# Reduction of Solar UV Radiation Due to Urban High-Rise Buildings – A Coupled Modelling Study

**DOI:** 10.1371/journal.pone.0135562

**Published:** 2015-08-11

**Authors:** Ka-Ming Wai, Peter K. N. Yu, Ka-Se Lam

**Affiliations:** 1 Department of Physics and Materials Science, City University of Hong Kong, Hong Kong, SAR, China; 2 Department of Civil and Environmental Engineering, The Hong Kong Polytechnic University, Hong Kong, SAR, China; University of Alabama at Birmingham, UNITED STATES

## Abstract

Solar UV radiation has both adverse and beneficial effects to human health. Using models (a radiative transfer model coupled to a building shading model), together with satellite and surface measurements, we studied the un-obstructed and obstructed UV environments in a sub-tropical urban environment featured with relatively high pollution (aerosol) loadings and high-rise buildings. Seasonal patterns of the erythemal UV exposure rates were governed by solar zenith angles, seasonal variations of aerosol loadings and cloud effects. The radiative transfer modelling results agreed with measurements of erythemal UV exposure rates and spectral irradiances in UVA and UVB ranges. High-rise buildings and narrow road width (height to width, H/W, ratios up to 15) reduced the modelled total UV (UVA+UVB) radiation and leave 10% of the un-obstructed exposure rate at ground-level at noon. No more than 80% of the un-obstructed exposure rate was received in the open area surrounded by 20-storey buildings. Our modelled reduction of UVB radiation in the urban environment was consistent with similar measurements obtained for Australia. However, our results in more extreme environments (higher H/W ratios) were for the first time reported, with 18% of the un-obstructed exposure rate remained at the ground-level center of the street canyon.

## Introduction

Solar UV radiation has both adverse and beneficial effects to human health. Harmful effects of the UVB (290–315 nm) radiation include sunburn, skin cancer, eye damage [1] but UVB radiation is also essential for vitamin D production [1–3]. Vitamin D production from UVB exposure is also related to body orientation [4–10]. The UVA (315–400 nm) radiation causes immunosuppression [11], skin aging [12] and melanoma [13]. Solar UV measurements are usually performed in open-areas [14–16] or made using satellite observations [17]. In urban areas such as those in Hong Kong, however, high-rise buildings or skyscrapers are located in close proximity. As such, humans at ground level frequently receive less UV radiation as compared to open areas due to the building shading effects. This results in inappropriate direct application of available results from previous studies to such unique environments. Recently, the measurements by McKinley et al. [18] have indicated that there was four times less of vitamin D produced within the urban environment. Pollution, temperature and humidity were not associated with vitamin D production.

The UV radiation received at the ground level depends on the solar azimuth angle. The UV levels are also affected by the atmospheric column ozone, the existence of clouds [15,16] and pollution levels in particular the aerosol concentrations [17,19,20]. Radiative transfer models have been used to estimate the UV radiation levels in open areas, taking the mentioned effects into consideration [21,22].

However, modelling studies are rarely found for the urban environment with high-rise buildings. Thus the first objective of the present work was to characterize the un-obstructed surface UV environment with an emphasis on the erythemal UV radiation in sub-tropical locations, which frequently suffered from (aerosol) pollution events and where relevant studies were seldom found. We then focused on the shading effects of high-rise buildings in terms of reduction in the UV radiation levels. This was achieved by using a radiative transfer model coupled to a building shading model.

## Materials and Methods

### Modelling and Observation of UV Radiation in Unobstructed Environment

A radiative transfer model—NCAR Tropospheric Ultraviolet and Visible Radiation (TUV) Model [23] was adopted to calculate the (direct and diffuse) UV-A, UV-B and erythema [24] exposure rates, with 8-stream discrete ordinate approach [25] and the effects from aerosols and clouds were taken into consideration. The TUV model has been widely used in various UV studies [22,26,27] and other relevant solar radiation studies [28–30]. The TUV model was run on days of every month in 2013 for model evaluation, when high daily global solar radiations (clear-sky condition) were recorded. For the model inputs, the column ozone and aerosol optical depth (AOD) at 388 nm were provided by the NASA’s Ozone Monitoring Instrument (OMI) Level 3 observations. Surface albedo was set to 0.1. The aerosol single scattering albedo was set as the model default.

The OMI observations for daily erythemal exposure rate and surface UV spectral irradiance at local noon time at 310 and 380 nm over Hong Kong in 2013 were used. The TUV predictions of erythema exposure rates were also compared with surface Brewer spectrophotometer measurements in a rural area of Hong Kong (Hok Tsui; 22.2°N, 114.3°E) [15].

### Modelling Urban Building Shading Effects

The TUV model was run on 21 June 2013 (summer solstice) with a clear-sky condition. The aerosol optical depth (AOD) of 0.2, as a background value observed by the OMI, was used unless otherwise specified. The reductions in solar radiation by building shades were modelled by a widely used commercial software—Autodesk Ecotect model, with the calculation details described by Ecotect [31]. Briefly, shading in the Ecotect model was determined using a shading mask which stored the percentage in shade for any surface. The model then calculated the solar radiation received at a particular surface by multiplying the unobstructed solar direct and diffuse radiation by the amount of shading at that surface. The building reflection effects were not taken into consideration as in other studies (see [32] and references therein). The TUV model predicted direct and diffuse UV-A and UV-B radiations which were used as inputs for the Ecotect model. The Ecotect model was set with four rows of high-rise buildings and roads in between. The outermost buildings had fixed heights of 20 storeys which were most typically found in the urban environment in Hong Kong but were built 3 decades ago or more. The inner two buildings “B1” and “B2” with heights (20, 40 and 60 storeys) varied with scenarios. The 40- and 60-storey scenarios represented typical building heights of recent establishments and some landmarks, respectively. We also varied the road widths (2, 4 and 6 driving lanes) for the various scenarios.

## Results and Discussion

### Characterization of Erythemal UV Radiation in Unobstructed Environment

**Fig 1:**
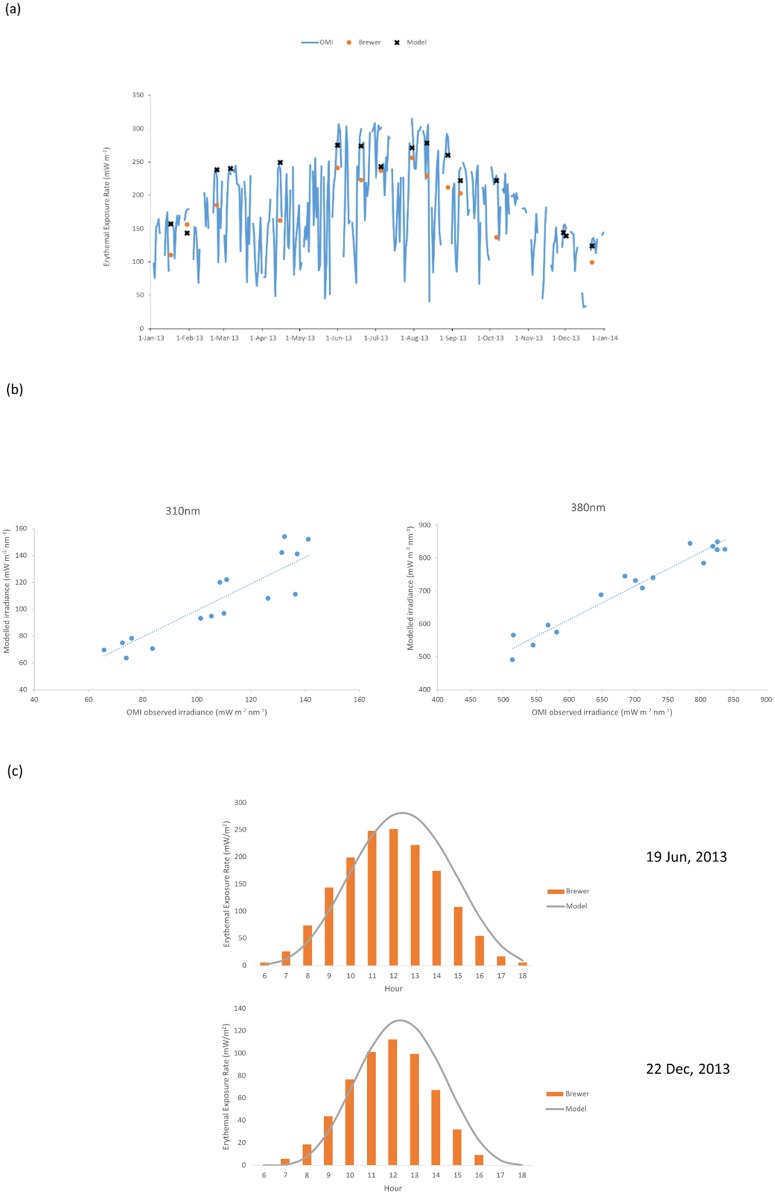
Comparison of measured and modelled UV. (a) Annual variation of the OMI noon-time erythermal exposure rates compared with the TUV modelling results and Brewer spectrophotometer measurements; (b) TUV modelled spectral irradiances (at 310 and 380 nm) compared with the OMI observations; and (c) modelled diurnal profile of erythemal exposure rate compared with the Brewer spectrophotometer measurements.

Fig 1a showed the annual variation of the OMI noon-time erythemal exposure rates, which was characterized with a summer maximum and a winter minimum in general. Except for the major role of solar zenith angles on the seasonal variation, the role of aerosol loadings in attenuating the UV radiation [13,14] was also crucial. For the latter, summer (winter) months featured with low (high) aerosol loadings due to the influence of clean marine air-mass and heavy rain scavenging (polluted continental outflow of pollution) [33,34]. An exception was the high aerosol loadings associated with approaching tropical cyclones in summer and autumn. These effects on building shading would be discussed in a following section. The summer (winter) maximum exposure rate was 300 (160) mW m^-2^, which was typical of a sub-tropical area [35]. The range of exposure rates in our study was within that obtained in other studies [14,17,36,37]. However, low exposures (e.g. 50 mW m^-2^), which occurred for the entire year, were attributed to the influence from the cloud covers. The ozone column measured by the OMI (figure not shown) was in the range 265–310 (200–255) DU from May to August (December to February) with a seasonal pattern similar to that of the erythemal exposure rate, instead of having an anti-correlation between each other. Therefore, the ozone column played an indirect or complicated role on the erythemal exposure rate for our study. Fig 1a also suggested that the exposure rates calculated by the TUV model agreed well with the OMI observations, while the surface-level Brewer spectrophotometer measurements tended to have low-biases. Fig 1b showed the agreement of the TUV modelled solar spectral irradiances at 310 nm (within UVB range) and 380 nm (within UVA range) with the OMI observations (r^2^ > 0.8). The modelled diurnal profile of erythemal exposure rate was also similar to that of the Brewer’s measurements (Fig 1c), suggesting good performance of the TUV model in solar UV calculations.

### Effects of Urban Shading on Solar UV Radiation

**Fig 2:**
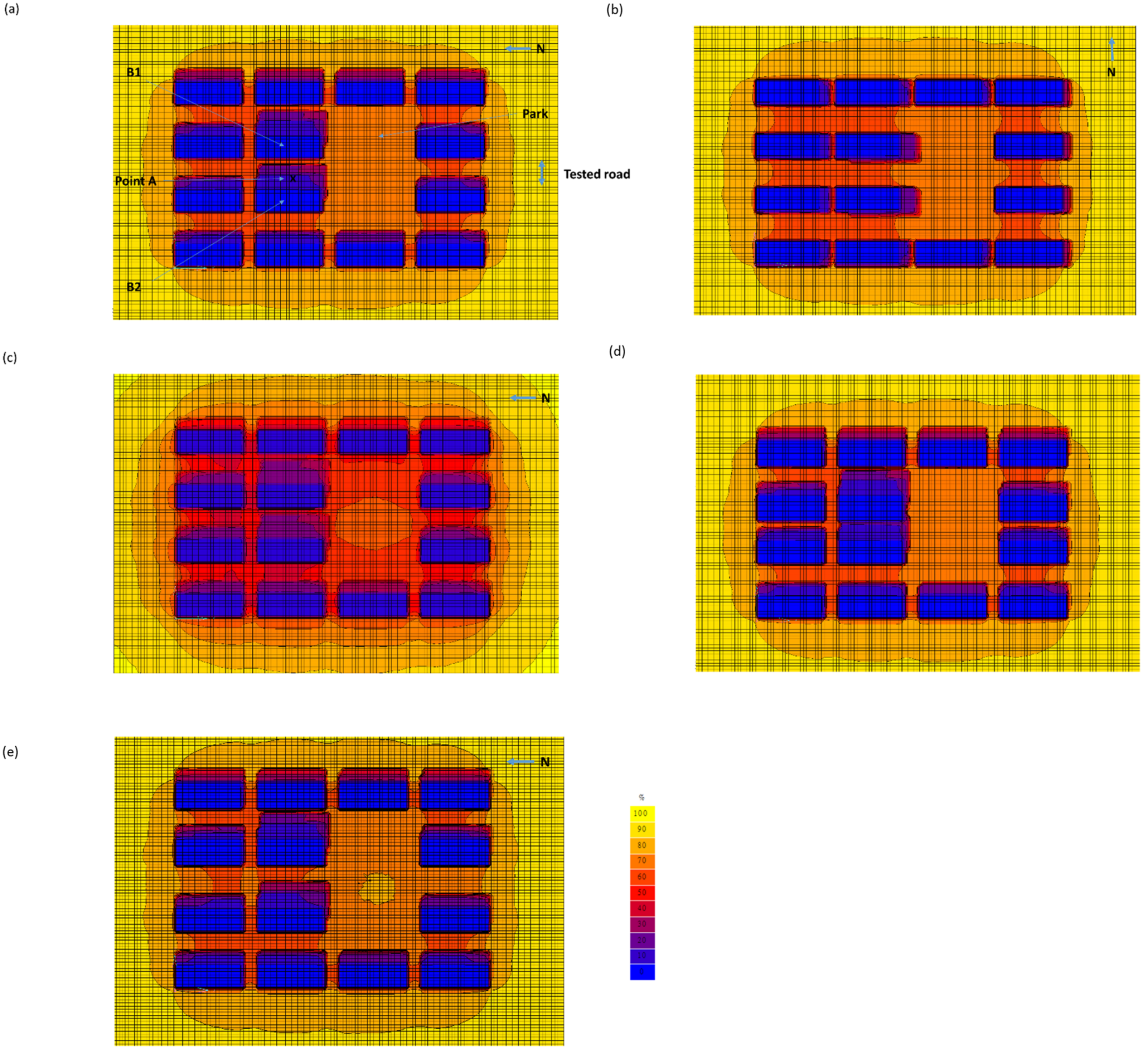
Urban shading effect scenarios – 40-storey building. (a) With 4 driving lanes; (b) same scenario as (a) but tilted at 90°; (c) same scenario as (a) but AOD = 0.75; (d) same scenario as (a) but with 2 driving lanes; and (e) same scenario as (a) but with 6 driving lanes.

The reduction in the ground-level total solar UV radiation (UVA + UVB) due to the shading effect of high-rise buildings was presented in terms of the ratio between the obstructed level and the unobstructed level (Fig 2a). The modelling results on total UV shown here can facilitate comparison with corresponding measurements made elsewhere [38,39]. It was observed that the results of total UV were similar to those for UVA, noting that the UVA exposure rates were 20–30 times larger than the UVB exposure rates. We focused on the scenarios in which the tested road had a south-north orientation with larger shading effects (Fig 2a) unless otherwise specified. The results were calculated at noon (12:00–13:00) when the exposure rate was the highest. The calculated diurnal profile of UVB exposure rate for unobstructed and obstructed scenarios would be discussed later.

The scenario consisting of a 40-storey building and a 4-lane road (height to width ratio: H/W = 6) was probably a most representative setting in the Hong Kong urban environment (Fig 2a). The park to the south of the two tested 40-storey buildings effectively received only 70% of the unobstructed radiation exposure rate. A maximum 60% of the radiation was received by most of the areas within the building arrays and further reductions (to less than 10%) were found within the shades of the tested buildings. When the road had an east-west orientation (Fig 2b), the radiation exposure rates were reduced rapidly in areas to the south of the tested buildings “B1” and “B2”. Since the sun was located approximately overhead of the buildings, fewer areas within the building array were in the building shadow (except those which were perpendicular to the test road) so they received 60% or more exposure rates. When the road had an orientation in between the mentioned cases (i.e., tilted at 45° anti-clockwise; figure not shown), the pattern of shaded areas was somehow between the two cases.

High aerosol loadings are generally associated with tropical cyclones approaching Hong Kong during the summer and autumn. Therefore, we performed a scenario with AOD of 0.75 as a typical value observed by the OMI for the scenario comprising the same settings of 40-storey building with a 4-lane road (Fig 2c). It was found that the decrease in radiation exposure rate was less rapid, especially within the areas of direct building shadows.

The exposure rate remained at 20% of the unobstructed rate within the shadows, in contrast to the 10% obtained for the similar scenario with AOD = 0.2. The difference was attributed to the decrease (by 57%) in the direct component of the radiation and to the increase (by 43%) in the diffuse component under the situation of high aerosol loadings.

For the scenario consisting of a 40-storey tested building and a 2-lane road (H/W = 10; Fig 2d), the areas between the tested buildings received less than 20% of the unobstructed exposure rate. About 70% of the other areas of the tested road between the 20-storey buildings received a similar exposure rate. In contrast, for the scenario of a 40-storey building with a 6-lane road (H/W = 4.3; fig 2e), the tested roads between the tested buildings and other areas of the tested road received higher exposure rates, with 60% between buildings and 70% near the park.

**Fig 3:**
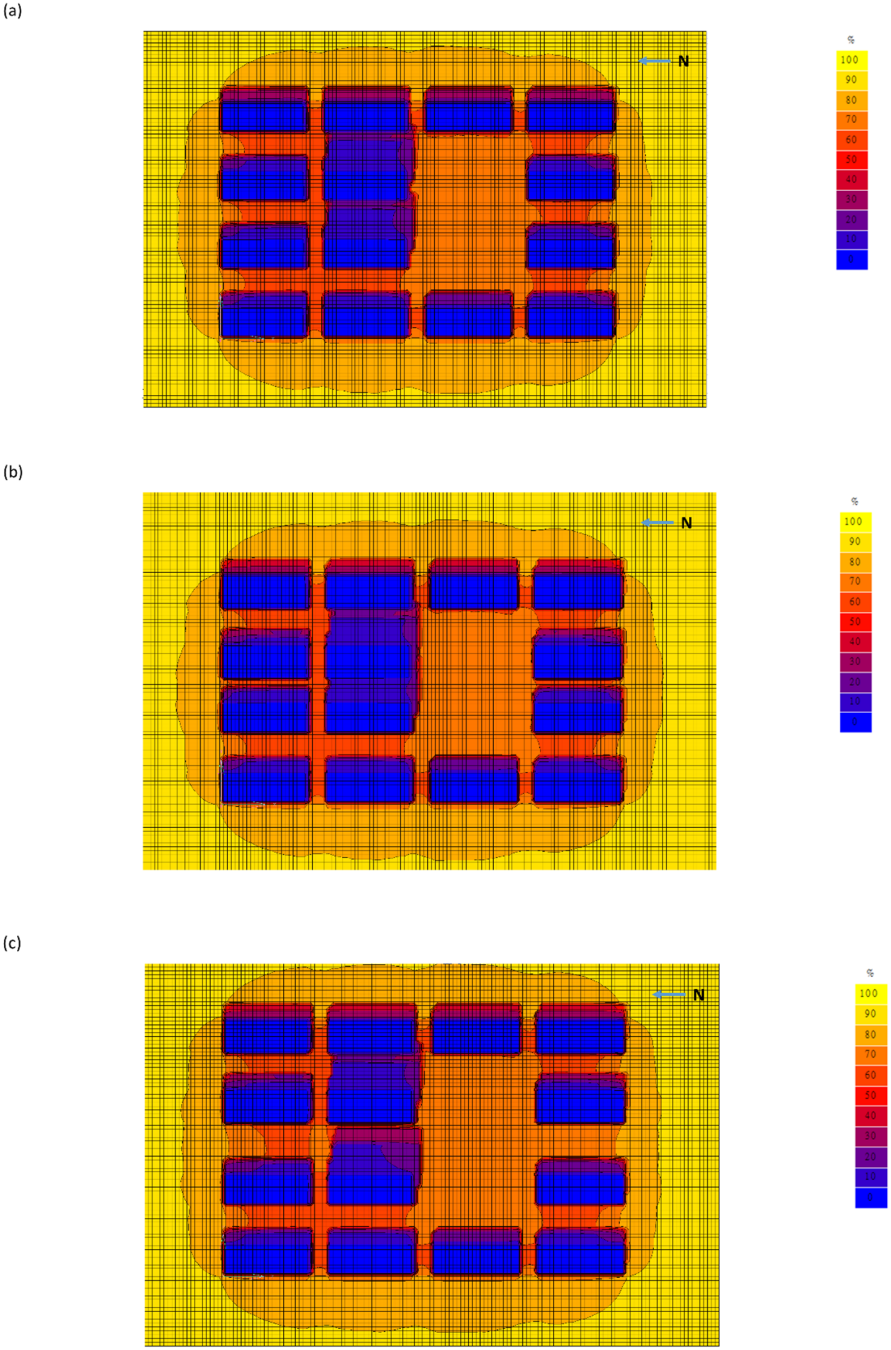
Urban shading effect scenarios – 60-storey building. (a) With 4 driving lanes; (b) same scenario as (a) but with 2 driving lanes; and (c) same scenario as (a) but with 6 driving lanes.

As regards the scenarios with taller tested buildings, the one comprising a 60-storey tested building and a 4-lane road (H/W = 9; Fig 3a) received no more than 20% of the exposure rate at the tested road within the tested buildings. For the extreme scenario comprising a 60-storey building with a 2-lane road (H/W = 15; Fig 3b), no more than 10% of the exposure rate at the tested road within the tested buildings was received. The exposure rate incident on the area was increased to 30% for the scenario comprising a 60-storey building with a 6-lane road (H/W = 6.4; Fig 3c).

**Fig 4:**
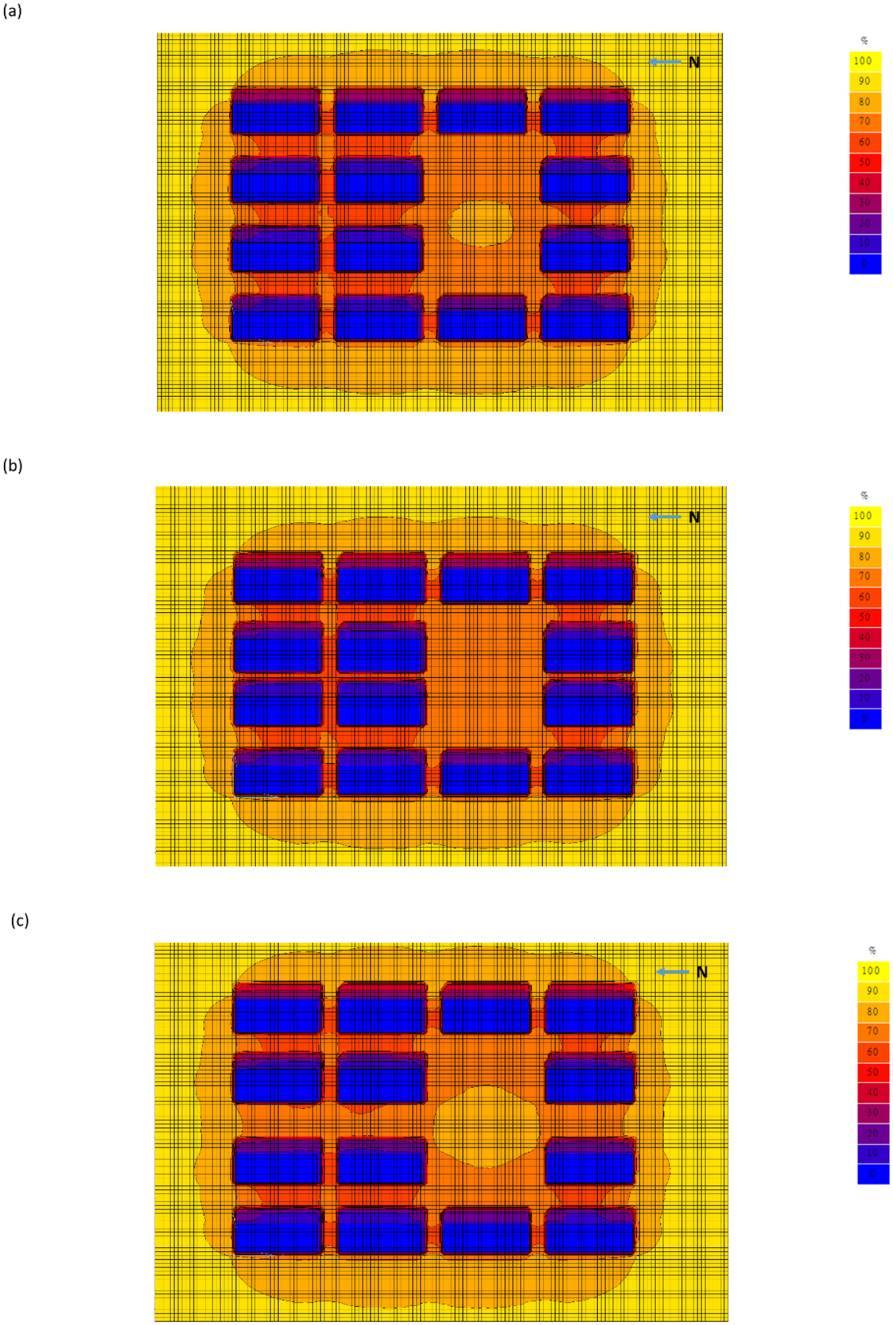
Urban shading effect scenarios – 20-storey building. (a) With 4 driving lanes; (b) same scenario as (a) but with 2 driving lanes; and (c) same scenario as (a) but with 6 driving lanes.

As regards the scenarios with lower tested buildings, for the one comprising a 20-storey tested building with a 4-lane road (H/W = 3; Fig 4a) about 40% of the tested road between the tested buildings received 60% of the unobstructed exposure rate, in contrast to the 10% for the tested road for the above scenario comprising a 40-storey building and a 4-lane road. A higher (80%) exposure rate was received by the tested road near the park. For the scenario comprising a 20-storey building and a 2-lane road (H/W = 5; Fig 4b), more than 70% of the tested road between the tested buildings received 20% of the exposure rate. For the scenario comprising a 20-storey building with a 6-lane road (H/W = 2.1; Fig 4c), more than 50% of the tested road between the buildings received 70% of the exposure rate. The areas receiving 80% exposure rate were enlarged to include the park itself and the nearby tested road.

**Fig 5:**
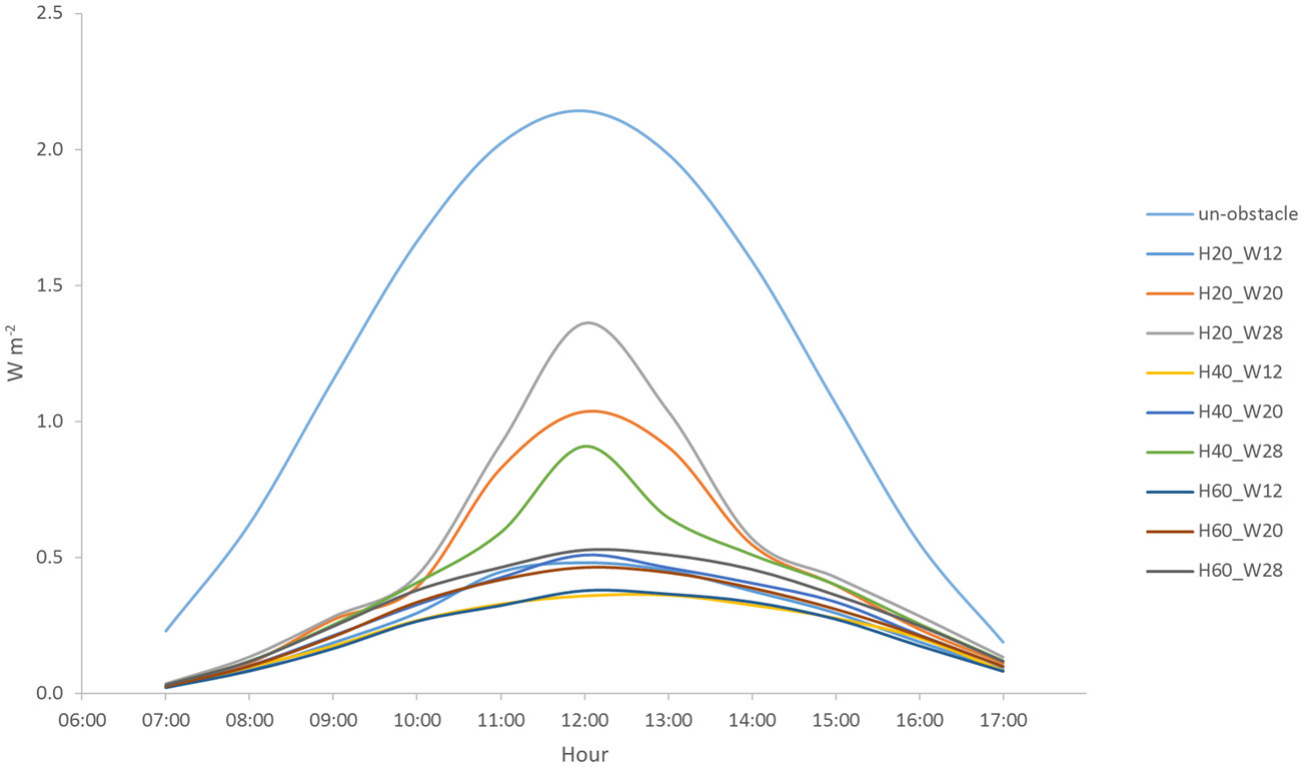
Diurnal profile. The calculated UVB radiation at Point A for the un-obstructed and various building shading scenarios.

As described above, vitamin D-deficiency was related to UVB radiation reduction in urban environments. Therefore, we also studied the diurnal profile of the calculated UVB radiation at Point A (Fig 2a) for the unobstructed scenario and scenarios with various building shadings (Fig 5). As expected, the reduction in UVB exposure rate due to building shading effects varied for different scenarios. The minimum reduction occurred for the scenario comprising a 20-storey building and a 6-lane road because of the relatively low building height and large road width, with 60% of the un-obstructed exposure rate. For the situation on another extreme, the scenario comprising a 60-storey building and a 2-lane road experienced the maximum reduction of UVB exposure rate and only 18% of the unobstructed exposure rate remained. McKinley et al. [18] measured a 25% of the total UV remained within an urban street canyon, which was commensurate with our results. A somewhat lower percentage obtained in our case was attributed to the higher H/W ratio.

## Conclusions

We have characterized the annual variation of erythemal UV exposure rates in sub-tropical urban unobstructed environments, which are influenced by air pollution, in particular high aerosol loadings. The erythemal UV, UVA and UVB results determined using a radiative transfer model were studied. The model was then coupled with an urban shading model to investigate the reduction of UV radiation in urban environments with high-rise buildings. The large attenuation in the UV radiation suggested that the results obtained from previous related studies performed in open-areas might not be applicable to the urban environments. Further studies are required to further quantify the effects of building shading on the solar UV radiation and the associated impacts on Vitamin D production, since the general public spends most of the daytime in such environments. The UVB radiation causes more sunburn than UVA radiation, according to the erythemal action spectrum [40]. Rayleigh scattering is more effective at short wavelengths while Mie scattering is not strongly wavelength dependent. Therefore, the effective sunburn radiation (UVB) might be more scattered in the atmosphere.
